# In vitro activity of selected antimicrobials against methicillin‐resistant *Staphylococcus pseudintermedius* of canine origin in Poland

**DOI:** 10.1002/vms3.1385

**Published:** 2024-03-28

**Authors:** Magdalena Kizerwetter‐Świda, Dorota Chrobak‐Chmiel, Ilona Stefańska, Ewelina Kwiecień, Magdalena Rzewuska

**Affiliations:** ^1^ Department of Preclinical Sciences Institute of Veterinary Medicine Warsaw University of Life Sciences Warsaw Poland

**Keywords:** antimicrobial resistance, disk diffusion, methicillin‐resistant *Staphylococcus pseudintermedius*, minimal inhibitory concentration, Poland

## Abstract

**Background:**

Methicillin‐resistant *Staphylococcus pseudintermedius* (MRSP) is an important veterinary pathogen. In general, only a few antimicrobials show in vitro activity against MRSP isolates.

**Objectives:**

The objective of this study was to determine the in vitro activity of selected antimicrobials, including last‐choice drugs, against clinical MRSP isolates of canine origin. The activity of 10 selected agents was evaluated against 41 clinical MRSP isolates.

**Methods:**

The disk diffusion method and minimal inhibitory concentration values were used for antimicrobial susceptibility testing (AST). The guidelines for staphylococci of canine or human origin were employed for the interpretation of the results.

**Results:**

Among the examined MRSP isolates, resistance to enrofloxacin and clindamycin was the most prevalent (*n* = 40; 97.6%). Resistance to doxycycline and gentamicin was observed in 83.0% (*n* = 34) and 68.3% (*n* = 28) of the isolates, respectively. Single isolates were resistant to chloramphenicol (*n* = 5; 12.2%) and rifampicin (*n* = 3; 7.3%), whereas all showed susceptibility to amikacin, vancomycin, mupirocin and linezolid. Predominantly, the results of AST obtained by both methods were consistent. Some discrepancies were observed for gentamicin; however, clinical breakpoints for staphylococci of human origin were used.

**Conclusions:**

Amikacin and chloramphenicol constitute potential treatment options in infections caused by MRSP and may be included in extended susceptibility testing in our geographical region. The determination of clinical breakpoints for some antimicrobials not incorporated in the recommendations should be a high priority in the veterinary diagnostics.

## Graphical abstract

The activity of 10 selected agents was evaluated against 41 clinical methicillin‐resistant *Staphylococcus pseudintermedius* (MRSP) isolates. The guidelines for staphylococci of canine or human origin were employed for the interpretation of the results. Lack of veterinary‐specific clinical breakpoints for some antimicrobials. Amikacin and chloramphenicol constitute potential treatment options in infections caused by MRSP and may be included in extended susceptibility testing in our geographical region.

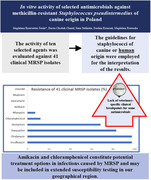

## 1 INTRODUCTION

Methicillin‐resistant *Staphylococcus pseudintermedius* (MRSP) strains are one of the most important opportunistic pathogens causing infections in dogs with diverse clinical manifestations, predominantly pyoderma, otitis externa, wound infections and urinary tract infections (UTI) (Loeffler & Lloyd, 2018; Lynch & Helbig, 2021). Sporadically, *S. pseudintermedius* may infect other species, mainly cats and humans (Kadlec et al., 2016; Ruscher et al., 2010; Wegener et al., 2018). MRSP strains are not susceptible to beta‐lactam antimicrobials and, often, show multidrug resistance (MDR). Therefore, the selection of effective therapy for infections caused by these bacteria represents a challenge for veterinarians (Loeffler & Lloyd, 2018; Moodley et al., 2014; Perreten et al., 2010). Furthermore, in some cases, only drugs classified by the World Health Organization (WHO) as critically important antimicrobials (CIAs) for human medicine show in vitro activity against MRSP (World Health Organization (WHO), 2019). The use of CIAs in veterinary medicine is controversial (Morris et al., 2017; Papich, 2021, 2023).

MRSP strains typically demonstrate susceptibility only to one or two, or even none of the antimicrobials routinely evaluated in a basic antibiogram. Thus, extended antimicrobial susceptibility testing (AST) must be performed, but these procedures are hampered by a lack of veterinary‐specific clinical breakpoints for some antimicrobials (Timofte et al., 2021). Moreover, there are no recommendations on which antimicrobials should be used in extended testing for MRSP. The primary goal of this study was to investigate the drug susceptibility status of MRSP isolates of canine origin in Poland. In addition, this work aimed to demonstrate the existence of discrepancies between minimal inhibitory concentration (MIC) and the disk diffusion test.

## 2 MATERIALS AND METHODS

### 2.1 MRSP isolates

The study was conducted on a collection of 41 MRSP isolates from clinical samples obtained from dogs submitted to the Microbiological Diagnostic Laboratory. The animals belonged to different owners and had no apparent epidemiological relationship. Clinical material for microbiological examination was collected according to the general principles. Data on the previous antimicrobial treatment in animals were not available. The dogs belonged to different breeds, 20 were female, 21 were male. Age ranged from 5 months to 15 years. Most of the clinical samples were collected from dermatological patients, summarized as skin and soft tissue infections (SSTI; *n* = 24; 58.5%). The second largest group consisted of urine samples from urinary tract infections (UTI; *n* = 7; 17.1%), followed by samples from respiratory tract infections (RTI; *n* = 6; 14.6%) and other samples (*n* = 4; 9.8%). Staphylococci were isolated in a routine bacteriological examination. The type of clinical materials from which the isolates were obtained is provided in Table S1. All isolates were stored at −20°C and subcultured for 24 h at 35°C on Columbia Agar supplemented with 5% sheep blood (Graso Biotech). The identification of *S. pseudintermedius* strains was based on standard bacteriologic methods and confirmed by the *nuc* PCR as described by Sasaki et al. (2010). Methicillin resistance was determined by the disk diffusion test with oxacillin (1 µg, Oxoid/Thermo Fisher Scientific) and confirmed using the *mecA* ne amplification according to the method provided by Strommenger et al. (2003).

### 2.2 Antimicrobial susceptibility testing of MRSP isolates

In vitro activity of 10 selected antimicrobials was evaluated using the disk diffusion method and the gradient diffusion method. The following antimicrobials were included in this study: enrofloxacin, clindamycin, doxycycline, amikacin, gentamicin, chloramphenicol, vancomycin, rifampicin, mupirocin and linezolid. Antimicrobial disk content and tested MIC ranges are disclosed in Table 1. The routine agar disk diffusion method was used for susceptibility testing in accordance with the CLSI M02 document (Clinical and Laboratory Standards Institute, 2018). MICs were determined by the gradient diffusion method using gradient strips (Liofilchem), according to the procedures determined by the manufacturer. Briefly, the isolates were suspended in sterile saline with a density of 0.5 in McFarland standard and spread on Mueller Hinton agar (bioMérieux). Subsequently, the antimicrobial disks and gradient strips were applied on the inoculated agar plates. After the routine incubation, the diameters of growth inhibition zones were measured, and MIC values were recorded. Where available, recommendations for staphylococci of canine origin listed in the CLSI VET01S document were used for results interpretation (Clinical and Laboratory Standards Institute, 2020). In case of the absence of veterinary‐specific criteria, human‐specific criteria for staphylococci from CLSI VET01S (Clinical and Laboratory Standards Institute, 2020) or EUCAST version 13.0 were applied (European Committee on Antimicrobial Susceptibility Testing (EUCAST), 2023). The interpretative criteria used for susceptibility testing of examined MRSP isolates are shown in Table 1. Quality control for both methods was performed using the following reference strains: *Staphylococcus aureus* ATCC 25923 for the disk diffusion method and *S. aureus* ATCC 33592 for MIC testing. The concentrations that inhibited 50% (MIC_50_) and 90% (MIC_90_) of the isolates were calculated for each antimicrobial. The isolates were evaluated for MDR using the criteria developed by Magiorakos et al. (2012). MDR was recognized when the isolates were interpreted as not susceptible to ≥1 agent in ≥3 antimicrobial categories. The scattergrams (Table S2) were designed by plotting zone diameters against corresponding MICs. Confidence intervals were calculated using the online sample size calculator tool (https://www.sample‐size.net).

**TABLE 1:** The interpretative criteria used for susceptibility testing of methicillin‐resistant *Staphylococcus pseudintermedius* isolates.

Antimicrobial	Interpretative criteria of inhibition zone diameter breakpoints (mm)			Interpretative criteria of MIC breakpoints (mg/L)			Disk content (µg)	The range of antimicrobial in gradient strips (mg/L)	Microorganism	Host	Recommendations
	S	I	R≥	S	I	R					
Enrofloxacin	≥23	17–22	≤16	≤0.5	1–2	≥4	5	0.002–32	*Staphylococcus* spp.	Dog	CLSI VET01S
Clindamycin	≥21	15–20	≤14	≤0.5	1–2	≥4	2	0.016–256	*Staphylococcus* spp.	Dog	CLSI VET01S
Doxycycline	≥25	21–24	≤20	≤0.12	0.25	≥0.5	30	0.016–256	*S. pseudintermedius*	Dog	CLSI VET01S
Amikacin	≥15^a^	^a^	<15^a^	≤4	8	≥16	30	0.016–256	*Staphylococcus* spp.	Dog	CLSI VET01S
Gentamicin	≥15	13–14	≤12	≤4	8	≥16	10	0.016–256	*Staphylococcus* spp.	Human	CLSI VET01S
Chloramphenicol	≥18	13–17	≤12	≤8	16	≥32	30	0.016–256	*Staphylococcus* spp.	Human	CLSI VET01S
Vancomycin	NA	NA	NA	≤2	4–8	≥16	NA	0.016–256	*S. aureus*	Human	CLSI VET01S
Rifampicin	≥20	17–19	≤16	≤1	2	≥4	5	0.002–32	*Staphylococcus* spp.	Human	CLSI VET01S
Mupirocin^b^	≥30	‐	<18	≤1		≥256	200	0.064–1024	*S. aureus*	Human	EUCAST 13.0
Linezolid	≥21	‐	<21	≤4		>4	10	0.016–256	*Staphylococcus* spp.	Human	EUCAST 13.0

Abbreviation: MIC, minimal inhibitory concentration.

^a^ EUCAST 13.0 breakpoints for *Staphylococcus aureus* of human origin.

NA, not applicable, disk diffusion method is not recommended for vancomycin and staphylococci.

^b^ Breakpoints for nasal *Staphylococcus aureus* decolonization and detection of a high level of mupirocin resistance.

## 3 RESULTS

### 3.1 MRSP isolates

Species‐specific PCR product of the *nuc* gene amplification of the expected size (926 bp) was obtained for all examined isolates (*n* = 41), thus all were identified as *S. pseudintermedius*. Moreover, all showed resistance in the disk diffusion test with oxacillin and the presence of *mecA* gene fragment of specific 532 bp length; therefore, they were recognized as MRSP.

### 3.2 Antimicrobial susceptibility testing of MRSP isolates

**FIGURE 1:**
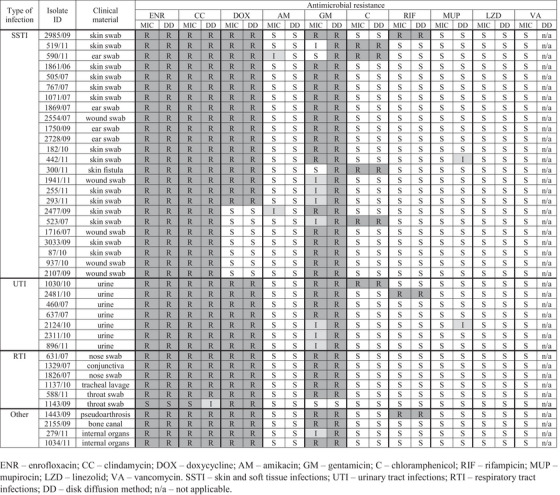
Heat map presenting the antimicrobial resistance profiles of 41 examined methicillin‐resistant *Staphylococcus pseudintermedius* clinical isolates obtained from dogs in Poland according to the type of infection. Each row represents one isolate. The results were obtained by minimal inhibitory concentration (MIC) determination and disk diffusion method. Dark grey fields represent resistant isolate, light grey – intermediate and white – susceptible.

The antimicrobial resistance profiles of examined MRSP isolates are presented in Figure 1. The scattergrams showing zone inhibition diameters and corresponding MICs are presented in Table S2. The MIC distributions and calculations of the MIC_50_ and MIC_90_ values are shown in Table 2. To simplify this table, the lowest MIC values from the gradient strips were included at a value less than or equal to 0.25 mg/L.

**TABLE 2:** The distribution of the minimum inhibitory concentrations (MICs), MIC_50_ and MIC_90_ values of 41 examined methicillin‐resistant *Staphylococcus pseudintermedius* clinical isolates obtained from dogs in Poland.

Antimicrobial	Number of isolates with the MIC value (mg/L)																			MIC_50_	MIC_90_	Resistant isolates (%)
	≤0.25	0.38	0.5	0.75	1	1.5	2	3	4	6	8	12	16	24	32	64	96	192	>256			
Enrofloxacin			1												40^a^					>32	>32	40 (97.6)
Clindamycin		1																	40	>256	>256	40 (97.6)
Doxycycline	7							2	1	3	12	14	2							8	12	34 (83.0)
Amikacin			1			6	15	12	5	1		1								2	4	0 (0)
Gentamicin	1	1					1	1		1	4	4	8	4	1	7	6	2		16	96	28 (68.3)
Chloramphenicol								9	21	6					4	1				4	32	5 (12.2)
Vancomycin	1			8	27	4		1												1	1.5	0 (0)
Rifampicin	38								1						2^a^					≤0.25	≤0.25	3 (7.3)
Mupirocin	40	1																		≤0.25	≤0.25	0 (0)
Linezolid	15	17	9																	0.38	0.5	0 (0)

*Note*: Grey fields represent the range of MIC values for resistant isolates.

^a^ —> the highest values tested 32 mg/L.

The highest resistance rates were observed for enrofloxacin and clindamycin, where all isolates except one were resistant (97.6%; CI95%: 87.1%–99.1%). Among 40 enrofloxacin and clindamycin‐resistant MRSP isolates, no inhibition zones were observed in the disk diffusion method. The MICs of both antimicrobials exceeded the highest values tested, 32 and 256 mg/L, accordingly. Only one isolate was recognized with the CLSI veterinary MIC clinical breakpoints as susceptible to these antimicrobials and by zone inhibition as susceptible to enrofloxacin and intermediate to clindamycin. The highest MIC_50_ and MIC_90_ values among MRSP isolates used in this study were observed for enrofloxacin and clindamycin, both exceeding 32 and 256 mg/L, respectively.

Doxycycline resistance was also widespread among examined MRSP isolates, found in 83.0% of isolates (CI95%: 68.0%–92.8%). The results obtained with disk diffusion were consistent with MIC values. Susceptibility and resistance were accurately recognized using the CLSI veterinary guidelines. All isolates classified as susceptible and resistant had the diameters of zone inhibition and MIC values within the range of corresponding clinical breakpoints. No errors (minor or major category) in susceptibility testing for doxycycline were observed. Resistant strains were characterized by MIC values within the range of 3–16 mg/L and zone diameters of 9–18 mm. For susceptible isolates, these values were no more or equal to 0.12 mg/L and 28–32 mm, respectively. MICs of doxycycline showed a bimodal distribution, with most of the isolates (*n* = 34) classified as resistant.

When the CLSI veterinary MIC guidelines for amikacin were applied, 40 examined isolates were classified as susceptible and 1 as intermediate. However, these recommendations do not include amikacin breakpoints for inhibition zone diameters. Therefore, EUCAST instructions for *Staphylococcus* spp. of human origin were used. All examined MRSP isolates were identified as susceptible to amikacin using disk diffusion. In general, MICs of amikacin were 0.5–4.0 mg/L and inhibition zones were 18–26 mm. Only the intermediate isolate had MIC 12 mg/L and an inhibition zone of 17 mm in diameter.

Most of the examined MRSP isolates showed gentamicin resistance. According to the MIC values, 28 isolates (68.3%; CI95%: 51.9%–81.9%) were assigned to the resistant, 9 to the intermediate and 4 to the susceptible category. Among two susceptible and nine intermediate isolates by MIC values, no inhibition zones were found. These discrepancies in the interpretation of the results were categorized as two major errors and nine minor errors. Considering only the results of the disk diffusion method, 39 isolates were identified as gentamicin‐resistant and only two as susceptible. It should be noted that the majority (*n* = 39) of MRSP tested showed no inhibition zones in the disk diffusion method. Of note, breakpoints for staphylococci of human origin were used. The bimodal MIC distribution was observed for gentamicin, affecting MIC_50_ and MIC_90_ values, which were 16 and 96 mg/L, respectively.

Five isolates recognized as chloramphenicol‐resistant by both methods (12.2%; CI95%: 4.0%–26.2%) showed high MICs (32–64 mg/L) and relatively small inhibition zones (10–11 mm in diameter). The remaining 36 examined MRSP isolates represented the most prevalent wild‐type population with a Gaussian model of MICs distribution in the range of 3–6 mg/L and inhibition zones of 20–28 mm in diameter. Therefore, MICs obtained for chloramphenicol displayed a bimodal distribution. However, it should be noted that human breakpoints were used for chloramphenicol.

Rifampicin resistance was confirmed in single isolates (*n* = 3; 7.3%; CI95%: 1.5%–19.9%). Three out of 41 examined MRSP isolates showed elevated MICs of rifampicin, including 2 with a value above 32 mg/L with no inhibition zones in disk diffusion and 1 with MIC 4 mg/L and an inhibition zone of 14 mm in diameter. The remaining 38 rifampicin‐susceptible isolates represented a wild‐type population with MIC values of no more or equal to 0.25 mg/L and large inhibition zones of 25–34 mm in diameter. Moreover, all examined strains showed susceptibility to vancomycin, mupirocin and linezolid characterized by low MIC values and large inhibition zones, typical for the wild‐type population. All MRSP isolates used for antimicrobial testing were recognized as multidrug‐resistant.

The antimicrobial resistance patterns of examined MRSP isolates according to the type of infection are shown in Fiureg 1. In general, the detected patterns were similar; resistance to between three and five antibiotics was observed in this study.

## 4 DISCUSSION

In recent years, the incidence of MSRP infections in small animal veterinary practice has increased (Lynch & Helbig, 2021). This is a worrying issue and, at the same time, it is a part of the global trend of increasing antimicrobial resistance. According to the guidelines of the World Association for Veterinary Dermatology, empirical antimicrobial treatment of infections caused by MRSP is contraindicated (Morris et al., 2017). Most of these strains are multidrug‐resistant, resulting in significantly limited treatment options (Papich, 2023; Perreten et al., 2010; Ruscher et al., 2010). Therefore, antimicrobials other than beta‐lactams are used for susceptibility testing and treatment. The problem is that veterinary‐specific clinical breakpoints for some of the crucial antimicrobials are not available. VetCAST and VAST, which are the EUCAST and CLSI sub‐committees for Veterinary AST, are constantly working on these guidelines. However, continuous surveillance of antimicrobial resistance as well as research on the distribution of MIC values is necessary.

Our results are consistent with literature data describing a high prevalence of MDR among MRSP (Hritcu et al., 2020; Kasai et al., 2016; Menadro, et al., 2019; Pires Dos Santos et al., 2016). Moreover, the analysis of the antimicrobial resistance patterns of MRSP isolates showed that MDR occurred regardless of the place of isolation of staphylococci. All isolates described in this study showed a high level of resistance to enrofloxacin, clindamycin and doxycycline, as observed in 97.6%, 97.6% and 83.0% of the isolates, accordingly. Resistance to clindamycin is also widespread in the world, reported in 66% of MRSP in the Netherlands and even 97% in Japan (Kasai et al., 2916; Wegener et al 2018). Tetracycline resistance in MRSP from the UK, Italy and Brazil is typically found in approximately 70% of the isolates, whereas only in 20% of MRSP in Argentine (Hritcu et al., 2020; Menadro, et al., 2019; Pires Dos Santos et al., 2016; Srednik et al. 2023). MRSP from different parts of the world are usually resistant to fluoroquinolones; resistance levels can vary from 52% of the isolates in the UK to even 97.6% in Slovenia and 98% in Japan (Hritcu et al., 2020; Menadro et al., 2019; Papić et al., 2021).

Gentamicin resistance in our study was relatively common, found in 68.3% of the strains. MICs of gentamicin showed a bimodal distribution, indicating that some of the isolates tested acquired resistance mechanisms. Importantly, we have observed inconsistency of the results obtained with the disk diffusion method and MICs evaluation, where 39 and 28 isolates were classified as resistant, accordingly. As noted, the results of two isolates (resistant by MICs) were categorized as major errors and nine (intermediate by MICs) as minor errors. These results were within acceptable error limits provided by CLSI. However, further analysis of isolates classified by MIC as susceptible or intermediate with no inhibition zones is planned. Moreover, it can be assumed that the application of recommendations developed for strains of human origin could have an impact on the interpretation of the results obtained. This confirms the critical need to develop clinical breakpoints for gentamicin and *S. pseudintermedius* isolated from dogs (Timofte et al., 2021). In other countries, gentamicin resistance is found with varying occurrence from 44% (The Netherlands) to 92.7% (Slovenia) of MRSP (Papić et al., 2021; Wegener et al., 2018).

Five isolates in our study had elevated MICs of chloramphenicol (12.2%) and three of rifampicin (7.3%), indicative of resistance, recognized with guidelines for human staphylococci. Occasional resistance to these two antimicrobials has been reported (Perreten et al., 2010; Ruscher et al., 2010). According to literature data, resistance to chloramphenicol was found less frequently in MRSP, ranging from 31% in UK and Slovenia (Hritcu et al., 2020; Papić et al., 2021) to 53% of the isolates in Japan (Kasai et al., 2016) of the strains tested. Interestingly, all of the MRSP strains studied in Argentina were susceptible to this antimicrobial (Gagetti et al., 2019). Single rifampicin‐resistant MRSP strains have been reported (Gagetti et al., 2019; Morais et al., 2023; Perreten et al., 2010; Wegener et al., 2018). Significantly different results were obtained in Brazil, where resistance to this antimicrobial was found in as many as 83% of MRSP strains (Viegas et al., 2022). One of the possible reasons for these discrepancies could be the occurrence of different MRSP clonal complexes in particular geographic regions of the world. It is known that MRSP strains belonging to different clonal complexes have specific drug susceptibility profiles (Phophi et al., 2023; Pires Dos Santos et al., 2016). Moreover, it may be related to distinct antimicrobial usage regimens in various countries.

When human‐specific breakpoints were used, all isolates in our study were recognized as susceptible to amikacin, vancomycin, mupirocin and linezolid with low MIC_50_ and MIC_90_ values, suggestive of wild‐type – MIC distribution. Nevertheless, the interpretation of the results obtained for amikacin was hampered by the fact that the CLSI veterinary recommendations include only breakpoints for MIC values, thus zone diameter breakpoints were applied from EUCAST (Clinical and Laboratory Standards Institute, 2020; EUCAST, 2023). There is a significant difference between the amikacin MIC breakpoints for susceptible strains in CLSI and EUCAST, that is 4 and 16 mg/L, respectively. Moreover, the EUCAST recommendations are dedicated to *S. aureus* of human origin and do not include the intermediate category. For this reason, the recognized susceptibility categories may be inaccurate. One isolate was identified as intermediate by MIC value but susceptible by growth inhibition zone diameter. This could be considered a minor error in interpretation, but only resistant or susceptible categorization could be obtained with EUCAST. When it comes to vancomycin, linezolid and mupirocin, despite the use of recommendations for staphylococci strains of human origin, the obtained inhibition zone diameters and MIC values were suggestive of a wild‐type population with full susceptibility. These results are consistent with the literature data (Bellato et al., 2022; Kasai et al., 2016; Wegener et al., 2018). However, previous reports describing uncommon cases of mupirocin resistance indicate a possibility of acquiring resistance if its genetic determinants become disseminated within the MRSP population (Kizerwetter‐Świda et al., 2021). Worryingly, Phophi et al. (2023) have detected linezolid resistance in one MRSP isolate in the United States.

As already mentioned in the introduction, some of the antimicrobials that may show in vitro activity against MRSP have been classified as CIAs (WHO, 2019). These include, among others vancomycin, linezolid, rifampicin and gentamicin, which should be used prudently in human and veterinary medicine (Prescott et al., 2021). Moreover, their use in animals is restricted by law in certain countries (European Medicines Agency [EMA], 2018). According to the guidelines of the International Society for Companion Animal Infectious Diseases (ISCAID), the use of antimicrobials like linezolid and vancomycin is strongly discouraged, as it is believed that they should be reserved for the treatment of serious human infections caused by methicillin‐resistant *S. aureus*. Nonetheless, these drugs can be used in exceptional cases when antimicrobials from the first and second tier (classification of antimicrobials given in the ISCAID guidelines) are not appropriate and show no in vitro activity (Hillier et al., 2014). Even if the use of CIAs is not prohibited by law, it remains controversial and may cause adverse side effects (Papich, 2023). However, antimicrobials approved for humans may be off‐label prescribed for animals in exceptional cases.

We are aware of the limitation of this study, which is employing only phenotypic methods used in AST. However, further studies determining the molecular basis of the resistance are planned. Moreover, we intended to get more data concerning the in vitro activity of selected antimicrobials against MRSP, especially when there are no veterinary recommendations.

## 5 CONCLUSION

This study highlights the need for harmonization of extended AST for MRSP isolates. To our best knowledge, such comparisons of the MIC values and corresponding results from the disk diffusion method concerning last‐choice antimicrobials, which may show activity against MRSP isolates, have never been published. Moreover, the results presented in this article indicate that amikacin and chloramphenicol may be therapeutic options for MRSP in our geographic region. In exceptional cases, mupirocin, linezolid and rifampicin may be considered potential therapeutic options and may be included in extended susceptibility testing.

## AUTHOR CONTRIBUTIONS

*Contributed to conceptualization and data curation; formal analysis and investigation; methodology; bacterial strains isolation; performed the experiments; analysed and interpreted the results; visualization; prepared the manuscript; tables and figures; writing – original draft; reviewing and editing*: Magdalena Kizerwetter‐Świda. *Contributed to conceptualization; methodology; and bacterial strains isolation; performed the experiments; analysed and interpreted the results; writing – reviewing and editing*: Dorota Chrobak‐Chmiel. *Contributed to writing – reviewing and editing*: Ilona Stefańska. *Contributed to writing – reviewing and editing*: Ewelina Kwiecień. *Contributed to conceptualization; methodology; and bacterial strains isolation; analysed and interpreted the results; writing – reviewing and editing*: Magdalena Rzewuska.

## CONFLICT OF INTEREST STATEMENT

There are no conflicts of interest to disclose.

## FUNDING INFORMATION

This research did not receive any specific grant from funding agencies in the public, commercial or not‐for‐profit sectors.

### PEER REVIEW

The peer review history for this article is available at https://publons.com/publon/10.1002/vms3.1385.

## ETHICS STATEMENT

No ethical approval was required as no animals were used.

## PLACE OF STUDY

The study was conducted at Microbiological Diagnostic Laboratory, Institute of Veterinary Medicine, Warsaw University of Life Sciences‐SGGW.

## Supporting information

Supporting Information

Supporting Information

## Data Availability

The data that support the findings of this study are available within the article, its supplementary materials and from the corresponding author upon reasonable request.
