# Supplementary material for: In vitro activity of selected antimicrobials against methicillin‐resistant Staphylococcus pseudintermedius of canine origin in Poland
Source: Vet Med Sci. 2024 Mar 28;10(3):e1385. doi: 10.1002/vms3.1385 (PMC10977695; doi:10.1002/vms3.1385)
Supplement: Supplementary file 1 — Supporting Information [file VMS3-10-e1385-s002.pdf]

Table S1. The characteristics of 41 methicillin-resistant *Staphylococcus pseudintermedius* clinical isolates obtained from dogs in Poland used in this study.

No.	Isolate id	Clinical material	Host
1	1861/06	skin swab	dog
2	460/07	urine	dog
3	505/07	skin swab	dog
4	523/07	skin swab	dog
5	631/07	nose swab	dog
6	637/07	urine	dog
7	767/07	skin swab	dog
8	1071/07	skin swab	dog
9	1329/07	conjunctiva swab	dog
10	1716/07	wound swab	dog
11	1826/07	nose swab	dog
12	1869/07	ear swab	dog
13	2554/07	wound swab	dog
14	1143/09	throat swab	dog
15	1443/09	pseudoarthrosis	dog
16	1750/09	ear swab	dog
17	2107/09	wound swab	dog
18	2155/09	femoral bone canal	dog
19	2477/09	skin swab	dog
20	2728/09	ear swab	dog
21	2985/09	skin swab	dog
22	3033/09	skin swab	dog
23	87/10	skin swab	dog
24	182/10	skin swab	dog
25	937/10	wound swab	dog
26	1030/10	urine	dog
27	1137/10	tracheal lavage	dog
28	2124/10	urine	dog
29	2311/10	urine	dog
30	2481/10	urine	dog
31	255/11	skin swab	dog
32	279/11	internal organs	dog
33	293/11	skin swab	dog
34	300/11	skin fistula	dog
35	442/11	skin swab	dog
36	519/11	skin swab	dog
37	588/11	throat swab	dog
38	590/11	ear swab	dog
39	896/11	urine	dog
40	1034/11	internal organs	dog
41	1941/11	wound swab	dog
