# Supplementary material for: In vitro activity of selected antimicrobials against methicillin‐resistant Staphylococcus pseudintermedius of canine origin in Poland
Source: Vet Med Sci. 2024 Mar 28;10(3):e1385. doi: 10.1002/vms3.1385 (PMC10977695; doi:10.1002/vms3.1385)
Supplement: Supplementary file 2 — Supporting Information [file VMS3-10-e1385-s001.pdf]

Table S2. Scattergrams showing the MICs values (mg/L) and growth inhibition zone diameters (mm) of antimicrobials tested in this study for 41 methicillin-resistant *Staphylococcus pseudintermedius* clinical isolates obtained from dogs in Poland. Horizontal lines indicate the MIC interpretative criteria (CLSI or EUCAST). Vertical lines correspond to the zone diameter interpretative criteria (CLSI or EUCAST) The grey background illustrates the intermediate category. The numbers in the scattergrams represent the numbers of isolates with a particular MIC and corresponding growth inhibition zone diameter.

a) Enrofloxacin

Enrofloxacin MIC (mg/L)	Enrofloxacin zone diameter (mm)																													
	≤6	7	8	9	10	11	12	13	14	15	16	17	18	19	20	21	22	23	24	25	26	27	28	29	30	31	32	33	34	35
>32	40																													
24																														
16																														
12																														
8																														
6																														
4																														
3																														
2																														
1.5																														
1																														
0.75																														
0.5																					1									
0.38																														
≤0.25																														

b) Clindamycin

Clindamycin MIC (mg/L)	Clindamycin zone diameter (mm)																													
	≤6	7	8	9	10	11	12	13	14	15	16	17	18	19	20	21	22	23	24	25	26	27	28	29	30	31	32	33	34	35
>256	40																													
192																														
96																														
64																														
32																														
24																														
16																														
12																														
8																														
6																														
4																														
3																														
2																														
1.5																														
1																														
0.75																														
0.5																														
0.38													1																	
≤0.25																														

c) Doxycycline

Doxycycline MIC (mg/L)	Doxycycline zone diameter (mm)																													
	≤6	7	8	9	10	11	12	13	14	15	16	17	18	19	20	21	22	23	24	25	26	27	28	29	30	31	32	33	34	35
>256																														
192																														
96																														
64																														
32																														
24																														
16				1			1																							
12					1	4	2	3	2	2																				
8						2	1		4	5																				
6					1	2																								
4						1																								
3										1			1																	
2																														
1.5																														
1																														
0.75																														
0.5																														
0.38																														
0.25																														
≤0.12																							1	1	1	1	3			

d) Amikacin

Amikacin MIC (mg/L)	Amikacin zone diameter (mm)																												
	≤6	7	8	9	10	11	12	13	14	15	16	17	18	19	20	21	22	23	24	25	26	27	28	29	30	31	32	33	34	35
>256																														
192																														
96																														
64																														
32																														
24																														
16																														
12												1																		
8																														
6																1														
4													1	1	2		1													
3														1	2	4	2	2		1										
2															2	3	7	2		1										
1.5														1	1	2	1	1												
1																														
0.75																														
0.5																					1									
0.38																														
≤0.25																														

e) Gentamicin

Gentamicin	Gantamicin zone dimeter (mm)																													
MIC (mg/L)	≤6	7	8	9	10	11	12	13	14	15	16	17	18	19	20	21	22	23	24	25	26	27	28	29	30	31	32	33	34	35
>256																														
192	2																													
96	6																													
64	7																													
32	1																													
24	4																													
16	6			1		1																								
12	4																													
8	4																													
6	1																													
4																														
3	1																													
2	1																													
1.5																														
1																														
0.75																														
0.5																														
0.38																1														
≤0.25																		1												

f) Chloramphenicol

Chloramphenicol	Chloramphenicol zone diameter (mm)																													
MIC (mg/L)	≤6	7	8	9	10	11	12	13	14	15	16	17	18	19	20	21	22	23	24	25	26	27	28	29	30	31	32	33	34	35
>256																														
192																														
96																														
64					1																									
32					3	1																								
24																														
16																														
12																														
8																														
6															1	1	3	1												
4															1	2	4	2		6	4		2							
3															2	3	1	2		1										
2																														
1.5																														
1																														
0.75																														
0.5																														
0.38																														
≤0.25																														

g) Rifampicin

Rifampicin MIC (mg/L)	Rifampicin zone diameter (mm)																													
	≤ 6	7	8	9	10	11	12	13	14	15	16	17	18	19	20	21	22	23	24	25	26	27	28	29	30	31	32	33	34	35
>32	2																													
24																														
16																														
12																														
8																														
6																														
4									1																					
3																														
2																														
1.5																														
1																														
0.75																														
0.5																														
0.38																														
≤0.25																				2	2	1	2	6	10	7	4	2	2	

h) Mupirocin

Mupirocin	Mupirocin zone diameter (mm)																													
MIC (mg/L)	≤6	7	8	9	10	11	12	13	14	15	16	17	18	19	20	21	22	23	24	25	26	27	28	29	30	31	32	33	34	35
>256																														
192																														
96																														
64																														
32																														
24																														
16																														
12																														
8																														
6																														
4																														
3																														
2																														
1.5																														
1																														
0.75																														
0.5																														
0.38																									1					
≤0.25																								2	13	16	6	3		

i) Linezolid

Linezolid	Linezolid zone diameter (mm)																														
MIC (mg/L)	≤6	7	8	9	10	11	12	13	14	15	16	17	18	19	20	21	22	23	24	25	26	27	28	29	30	31	32	33	34	35
>256																														
192																														
96																														
64																														
32																														
24																														
16																														
12																														
8																														
6																														
4																														
3																														
2																														
1.5																														
1																														
0.75																														
0.5																					2	5	2							
0.38																					2	2	8	4	1					
≤0.25																				1	2	5	2	3	2					
